# Decision regret after reirradiation of the primary site in patients with prostate cancer

**DOI:** 10.1016/j.ctro.2025.101019

**Published:** 2025-07-19

**Authors:** Alexander Fabian, Bilgesu Sahin Öztürk, Lars Haack, Severin Rodler, Christof van der Horst, Christian Schulz, Claudia Schmalz, Stefan Huttenlocher, Olaf Wittenstein, Oliver Blanck, Frank-André Siebert, David Krug

**Affiliations:** a Department of Radiation Oncology, University Hospital Schleswig-Holstein Campus Kiel, Kiel, Germany; b Saphir Radiosurgery Center Northern Germany, Kiel, Germany; c Department of Urology, University Hospital Schleswig-Holstein Campus Kiel, Kiel, Germany; d URODOCK Urology Group Practice, Kiel, Germany; e Department of Radiotherapy and Radiation Oncology, University Medical Center Hamburg-Eppendorf, Hamburg, Germany

**Keywords:** Prostate cancer, Reirradiation, Radiotherapy, Brachytherapy, Stereotactic body radiotherapy, SBRT, Decision regret, Patient-reported outcome, Health-related quality of life

## Abstract

**Purpose:**

A subset of prostate cancer patients develops local relapse at the primary site after radiotherapy. The optimal local salvage strategy is unknown. Therefore, we aimed to explore prevalence and determinants of decision regret among patients after reirradiation of the primary site.

**Materials and methods:**

We surveyed 31 patients in a cross-sectional bi-centre exploratory study. Reirradiation was high dose-rate brachytherapy (HDR-BT) in 21 and stereotactic body radiotherapy (SBRT) in 10 patients. Decision regret (DR) was measured using the Decision Regret Scale (DRS) (range: 0–100; higher values higher regret). Further patient-reported outcomes (PRO) included the EPIC-26, EORTC QLQ-C30, PRO-CTCAE, and PSCC questionnaires. Univariable associations of decision regret and potential determinants were assessed by one-way ANOVA or Pearson’s correlation.

**Results:**

Median age at reirradiation was 75 years. Median time intervals from initial radiotherapy to reirradiation was 8 years and 4 years from reirradiation to survey. The mean DRS score was 10 (SD: 14). No (0 points), mild (1–25 points), or strong regret (>25 points) was reported by 45 % (14/31), 48 % (15/31), and 7 % (2/31) of the patients, respectively. DR was significantly associated with PRO of urinary symptom burden and toxicity as well as levels of shared-decision making and patient satisfaction. HDR-BT vs. SBRT, further local relapse, and progression-free survival were not associated with DR.

**Conclusions:**

DR was mild among prostate cancer patients after reirradiation to the primary site. PRO on symptom burden and shared decision making may be associated with DR. These findings should be validated and may inform treatment decisions on local salvage therapy.

## Highlights

• The optimal local treatment strategy for radiorecurrent prostate cancer is unknown.
• We analyzed decision regret among 31 patients after reirradiation via HDR-BT or SBRT.
• Most patients had either no or mild levels of decision regret.
• PRO on symptoms, toxicity, SDM and satisfaction were associated with regret.

## 1 Introduction

Various effective treatment options exist for patients with localized prostate cancer, often including some form of radiotherapy [1]. However, local relapse after radiotherapy to the primary site plays an increasingly important role in clinical practice as PSMA-PET/CT now allows for localization of the radio-recurrent site in case of biochemical failures [2]. These radio-recurrent local relapses may either affect patients treated with primary radiotherapy or patients that were treated with adjuvant or salvage radiotherapy to the prostate fossa after initial surgery. For example, a recent *meta*-analysis of patients treated with primary radiotherapy in clinical trials reported that 13 % of high risk and 7 % of intermediate risk patients experienced local relapse at a median follow-up of 11 years, respectively [3]. Further, local relapse was associated with worse subsequent oncological outcomes [3]. In addition, local relapse has been shown to be the most common site of failure after primary radiotherapy [4]. The management of patients with isolated radio-recurrent local relapse is not standardized. Besides systemic therapy, there are various local treatment options including surgery, high intensity focused ultrasound, and reirradiation with brachytherapy or stereotactic body radiotherapy (SBRT) [5,6]. As there are no randomized trials to guide selection of local therapy in radio-recurrent disease and as patients still often face a prognosis of living many further years, patient’s decision regret after pursuing local therapy of radio-recurrent disease is informative.

Decision regret from a patient’s view relates to an unpleasant feeling with regard to a healthcare decision. Chehade and colleagues define decision regret in a concept analysis as “*a negative cognitive-emotional phenomenon experienced following a health-related decision in an immediate or delayed occurrence*” [7]. Decision regret is captured by patient-reported outcome measures (PROM) such as the Decision Regret Scale (DRS) that ranges from 0 to 100 with higher scores indicating stronger regret [8]. For example, a systematic review of decision regret reported a mean DRS score of 16.5 in patients with various health conditions [9]. As Chehade and colleagues further elaborate “*regret relates to the decision-making process, treatment options, and/or outcomes and results in negative and positive consequences*” [7]. Various potential factors may hence influence the extent of decision regret. These factors include but are not limited to oncological outcomes, health-related quality of life (HRQoL), toxicity, levels of shared-decision making, or overall patient satisfaction [9,10].

No studies, to our knowledge, have yet reported on decision regret from the patient’s view after reirradiation as a whole and after reirradiation of radio-recurrent relapses at the primary site in patients with prostate cancer. This knowledge could help in clinical decision-making and patient discussion. Therefore, we conducted an exploratory study with the primary objectives to investigate the prevalence and determinants of decision regret in prostate cancer patients treated with high dose-rate brachytherapy (HDR-BT) or SBRT for radio-recurrent relapse at the primary site.

## 2 Materials and methods

### 2.1 Study design

We conducted an exploratory cross-sectional bi-centre study in two participating centres including prostate cancer patients treated with reirradiation to the primary site. Patients were treated either with HDR-BT at the Department of Radiotherapy, University-Hospital Schleswig-Holstein in Kiel, Germany or with SBRT at the Saphir Radiosurgery Centre Northern Germany in Kiel/Güstrow, Germany. Patient inclusion criteria were: i.) history of prostate cancer, ii.) previous radiotherapy to the primary site, iii.) reirradiation to the primary site either with HDR-BT or SBRT as salvage treatment at least six months ago, iv.) available reirradiation treatment plans, v.) ability to understand and complete questionnaires, vi) patient age > 18 years, and vii.) written informed consent. Potentially eligible patients were identified in local electronic health records and offered study participation. Paper-based patient-reported outcomes were then sent to patients and re-sent via post. The local ethical committee provided ethical approval prior to enrolment of the first patient (Kiel, Germany D505/23). The study was preregistered on the Open Science Framework (https://doi.org/10.17605/OSF.IO/A6DC3, „cohort E“) and analyses of a different cohort within this study protocol have been published previously [11,12].

### 2.2 Outcomes and variables

The primary outcome of this study was decision regret based on the PROM DRS [8]. The DRS comprises five items related to regret from the patient’s view: i.) “It was the right decision”, ii.) “I regret the choice that was made”, iii.) “I would go for the same choice if I had to do it over again”, iv.) “The choice did me a lot of harm”, and v.) “The decision was a wise one”. All items are framed on a 5-point Likert scale. To compute the DRS summary score, both negatively framed items are reversed. Then each question ranging from 1 to 5 is subtracted by 1 and multiplied by 25. The total score is calculated from the mean score of all five items. The summary score ranges from 0 to 100 with higher values indicating higher regret. We classified levels of regret in “no decision regret” (0 points), “mild decision regret” (1–25 points), and “strong decision regret” (>25 points) [8,13].

Additional patient-reported outcome measures included the European Organization for Research and Treatment of Cancer Quality of Life Group-C30 (EORTC QLQ-C30), Expanded Prostate Cancer Index Composite (EPIC-26), Patient-reported Outcome Version of the Common Terminology Criteria of Adverse Events (PRO-CTCAE), Patient Satisfaction with Cancer-related Care (PSCC), and Self-Administered Comorbidity Questionnaire (SCQ) [[14], [15], [16], [17], [18]]. The EORTC QLQ-C30 is a cancer-related generic questionnaire on HRQoL consisting of 30 items. Higher scores of its symptom scales indicate worse symptoms and higher scores of its functioning scales indicate better functioning [14]. The EPIC-26 covers domains of HRQoL relevant to patients with prostate cancer [15]. These domains include: urinary incontinence, urinary irritative/obstructive, bowel (“overall gastrointestinal function”), sexual, hormonal, and a single question on overall urinary function. Higher scores reflect better functioning. PRO-CTCAE capture patient-reported toxicity [16]. We surveyed PRO-CTCAE domains on diarrhoea, abdominal pain, faecal incontinence, painful urination, urinary urgency, urinary frequency, and urinary incontinence. These PRO-CTCAE domains are ranked on a 5-point Likert scale and may be subdivided by items of frequency, severity, and daily interference. To combine these items to a single score for each domain, we applied composite grading as described by Basch and colleagues [19]. PRO-CTCAE composite grades range from zero to three and a higher score indicates higher toxicity. The PSCC is a questionnaire on patient satisfaction including 18 5-point Likert scaled items [17]. We used “question 3 (“I felt included in decisions about my health“) as surrogate for shared-decision making and also calculated a summary score across all 18 items for overall patient satisfaction. The score ranges from 0 to 100 and higher scores reflect higher patient satisfaction. The SCQ captures patient-reported comorbidity counting relevant comorbidities and the need for their treatment as well as medication [18].

We extracted patient, disease, and treatment characteristics from electronic health records and consulted primary physicians for supplementary follow-up data. Initially we planned to accumulate radiation doses to organs at risk, but this was deemed infeasible because of limited availability of initial radiation plans.

### 2.3 Statistical analysis

We used descriptive statistics to describe the study cohort and decision regret. Oncological outcomes were calculated by Kaplan-Meier estimators from the date of the last fraction of reirradiation until the occurrence of a respective event or last follow-up. Progression-free survival (PFS) combined biochemical relapse as per Phoenix-criterion (i.e. PSA 2ng/ml above nadir), local relapse, and distant relapse from reirradiation until study participation or last available follow-up. Patient death was not considered for PFS, as all patients were alive at study participation. We investigated associations of decision regret and covariables with Pearson’s correlation or one-way Analysis of Variance (ANOVA) depending on the variable’s scale. We did not perform multivariable analyses due to the limited sample size. Further, we did not correct for multiple testing in light of the exploratory setting of our study [20]. A two-sided p-value < 0.05 was considered statistically significant. All analyses were performed using JASP v0.19.3 (JASP Team [2024], Amsterdam, the Netherlands).

## 3 Results

### 3.1 Patient and treatment characteristics

Among 47 patients who were treated with reirradiation to the primary site for prostate cancer, 31 were reached and consented to participate as shown in Supplementary Fig. S1. Of these, 68 % (21/31) received HDR-BT and 32 % (10/31) received SBRT at reirradiation, respectively.

Patient and treatment characteristics of the initial radiotherapy course are shown in Table 1 for the total cohort and subdivided into patients who later received HDR-BT reirradiation or SBRT reirradiation. At the initial radiotherapy and in the total cohort, the median age was 65 years and 58 % (18/31) of the patients had high risk disease according to the D’Amico risk groups. The initial course of radiotherapy was postoperative EBRT after radical prostatectomy in 19 % (6/31) of the patients.

**Table 1:** Patient (n = 31) and treatment characteristics at initial radiotherapy.

**Patient characteristics at initial therapy**				
		**HDR-BT reirradiation** **group 68 % (n = 21)**	**SBRT reirradiation** **group 32 % (n = 10)**	**Total** **100 % (n = 31)**
Age (years)		Median: 62; IQR: 9	Median: 62; IQR: 9	Median: 65; IQR: 10
iPSA (ng/ml)	≤10	48 % (10)	50 % (5)	48 % (15)
	>10	19 % (4)	10 % (1)	16 % (5)
	>20	24 % (5)	40 % (4)	29 % (9)
D'Amico risk group	Low	28 % (6)	20 % (2)	26 % (8)
	Intermediate	10 % (2)	20 % (2)	13 % (4)
	High	57 % (12)	60 % (6)	58 % (18)
T stage	T1	24 % (5)	40 % (4)	29 % (9)
	T2	48 % (10)	50 % (5)	48 % (15)
	T3	28 % (6)	10 % (1)	22 % (7)
**Treatment characteristics at initial radiotherapy**				
Initial radiotherapy regimen				
Primary EBRT		14 % (3)	30 % (3)	19 % (6)
	40-44x1.8 Gy	9 % (2)	20 % (2)	12 % (4)
	36x2Gy	5 % (1)	0 % (0)	3 % (1)
	5x7Gy (SBRT)	0 % (0)	10 % (1)	3 % (1)
Primary EBRT & HDR-BT boost	20-28x1.8–2 Gy EBRT & 2x 15/8Gy* HDR-BT	38 % (8)	50 % (5)	42 % (13)
Primary LDR-Brachytherapy	145 Gy	24 % (5)	10 % (1)	19 % (6)
Postoperative EBRT after RP		24 % (5)	10 % (1)	19 % (6)
	33x2Gy	14 % (3)	10 % (1)	13 % (4)
	37x1.8 Gy	5 % (1)	0 % (0)	3 % (1)
ADT	Yes	14 % (3)	30 % (3)	19 % (6)
	No	86 % (18)	70 % (7)	81 % (25)
ADT duration (months)	Long (>6)	14 % (3)	20 % (2)	16 % (5)
	Short (≤6)	0 % (0)	10 % (1)	3 % (1)

Numbers may not add up to 100 % due to rounding errors or missing values. Abbreviations: ADT, Androgen deprivation therapy; EBRT, External beam radiation therapy; HDR-BT, High dose-rate brachytherapy; IQR, Interquartile range; LDR, Low dose rate; SBRT, Stereotactic body radiotherapy; SD, Standard deviation; SIB, simultaneously integrated boost. * 15 Gy HDR-BT prescribed to the peripheral prostatic zone and 8 Gy to the whole prostate.

Patient characteristics at and treatment characteristics of the course of reirradiation are shown in Table 2. Concerning the whole cohort at reirradiation, the median age was 75 years and the median time interval from initial radiotherapy to reirradiation was 8 years. Median PSA-doubling time prior to reirradiation was 18 months. Most patients (97 %; 30/31) had a PSMA-PET/CT prior to reirradiation while a minority of the patients had a repeat biopsy (26 %; 8/31) or MRI (26 %; 8/31) prior to reirradiation. Treatment concepts of reirradiation varied. Among those patients who received HDR-BT as reirradiation, all patients were treated with an Ir-192 source and endorectal ultrasound-based planning at one weekly fraction by analogy to a prior publication from the treating centre [21]. All HDR-BT reirradiation patients after initial primary radiotherapy were treated to the whole prostate. Patients who received SBRT as reirradiation were treated every other day (n = 8) or on consecutive workdays (n = 2) on a CyberKnife platform with implanted fiducials. Among these, the majority of the patients received reirradiation to the intraprostatic recurrence only, i.e. without coverage of the whole prostate.

**Table 2:** Patient characteristics (n = 31) at and treatment characteristics of reirradiation.

**Patient characteristics at reirradiation**				
		**HDR-BT reirradiation** **group 68 % (n = 21)**	**SBRT reirradiation** **group 32 % (n = 10)**	**Total** **100 % (n = 31)**
Age (years)		Median: 75; IQR: 8	Median: 75; IQR: 13	Median: 75; IQR: 8
Performance status (ECOG)	0	19 % (4)	10 % (1)	16 % (5)
	1	57 % (12)	90 % (9)	68 % (21)
	2	24 % (5)	0 % (0)	16 % (5)
Interval (years) of primary radiotherapy to reirradiation		Median: 10.8; IQR: 7.1	Median: 6.4; IQR: 4.7	Median: 8.0; IQR: 5.9
PSA (ng/ml)		Mean: 6.3; SD: 3.9	Mean: 6.3; SD: 4	Mean: 6.3; SD: 3.8
PSA-Doubling time (months)		Median: 22; IQR: 12	Median: 11; IQR: 8	Median: 18; IQR: 14
T stage	T1	76 % (16)	90 % (9)	80 % (25)
	T2	10 % (2)	10 % (1)	10 % (3)
	T3	5 % (1)	0 % (0)	3 % (1)
PET-CT staged	Yes	95 % (20)	100 % (10)	97 % (30)
	No	5 % (1)	0 % (0)	3 % (1)
Confirmatory biopsy	Yes	29 % (6)	20 % (2)	26 % (8)
	No	71 % (15)	80 % (8)	74 % (23)
MRI staged	Yes	14 % (3)	50 % (5)	26 % (8)
	No	86 % (18)	50 % (5)	74 % (23)
Prostate volume (cm3)		Median: 24; IQR: 9	Median: 39; IQR: 12	Median: 27; IQR: 17
On ADT	Yes	29 % (6)	20 % (2)	26 % (8)
	No	71 % (15)	80 % (8)	74 % (23)
**Treatment characteristics of reirradiation**				
HDR-BT		68 % (21)	−	−
Whole prostate	3x10Gy	14 % (3)	−	−
Whole prostate & SIB on intraprostatic recurrence	3x8-8.5 Gy & 3x10Gy (SIB)	62 % (13)	−	−
Prostate fossa recurrence (after RP)	3x10Gy	24 % (5)	−	−
SBRT		−	32 % (10)	−
Whole prostate	5x6Gy (75 % IDL)	−	20 % (2)	−
Intraprostatic recurrence only	3x8-10 Gy (70–81 % IDL)	−	60 % (6)	−
Intraprostatic recurrence only	5x6Gy (65 % IDL)	−	10 % (1)	−
Prostate fossa recurrence (after RP)	3x7Gy (75 % IDL)	−	10 % (1)	−

Numbers may not add up to 100% due to rounding errors or missing values. Abbreviations: ADT, Androgen deprivation therapy; EBRT, External beam radiation therapy; HDR-BT, High dose-rate brachytherapy; IDL, Isodose line; IQR, Interquartile range; LDR, Low dose rate; RP, Radical prostatectomy; SBRT, Stereotactic body radiotherapy; SD, Standard deviation; SIB, Simultaneous integrated boost.

At survey and in the whole cohort, the median age was 81 years (IQR: 9 years) (Supplementary Table S1). Patients had a median of 5 patient-reported comorbidities (IQR: 5). Mean global HRQoL per EORTC QLQ-C30 was 65 (SD: 25). The median time interval from reirradiation to survey, and hence median follow-up, was 45 months (IQR: 60 months). Relevant patient-reported outcomes of the EPIC-26, EORTC QLQ-C30, and PSCC are shown in Supplementary Table S2.

### 3.2 Oncological outcomes of reirradiation

**Fig. 1:**
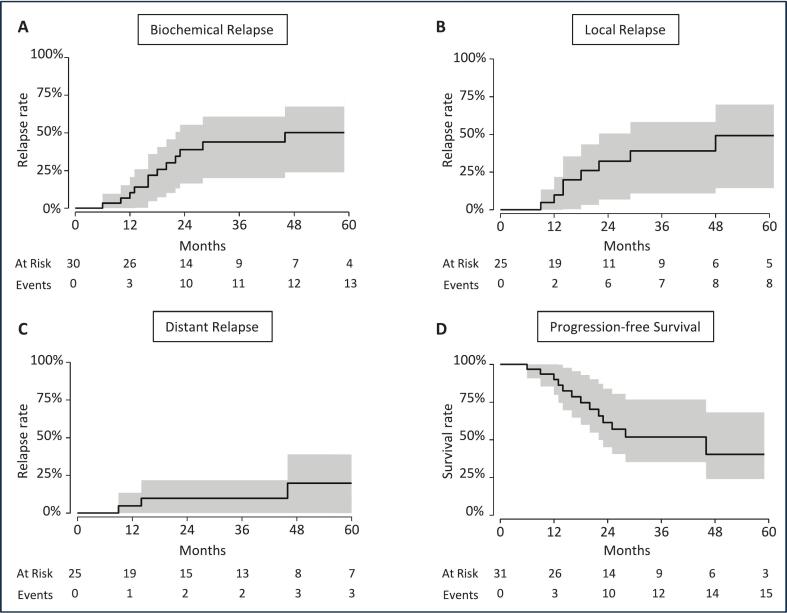
Oncological outcomes of participating prostate cancer patients (n = 31) after reirradiation of the primary site. Kaplan-Meier estimators are shown with corresponding 95 % confidence intervals. Panel A, B, C, and D show biochemical relapse, local relapse, distant relapse, and progression-free survival rates, respectively.

Fig. 1 shows oncological outcomes after reirradiation of all participating patients. Biochemical relapse rates at 1 year and 3 years were 10.3 % (95 %-CI: 0 – 20.6 %) and 43.9 % (95 %-CI: 19.9 – 60.7 %), respectively. Local relapse rates at 1 year and 3 years were 9.8 % (95 %-CI: 0 – 21.8 %) and 38.9 % (95 %-CI: 10.8 – 58.2 %), respectively. Distant relapse rates at 1 year and 3 years were 4.8 % (95 %-CI: 0 – 13.4 %) and 9.8 % (95 %-CI: 0 – 21.8 %), respectively. Progression-free survival at 1 year and 3 years was 90 % (95 %-CI: 79.8 % – 100 %) and 51.6 % (95 %-CI: 35.1 – 76.7 %), respectively.

### 3.3 Toxicity after reirradiation

**Fig. 2:**
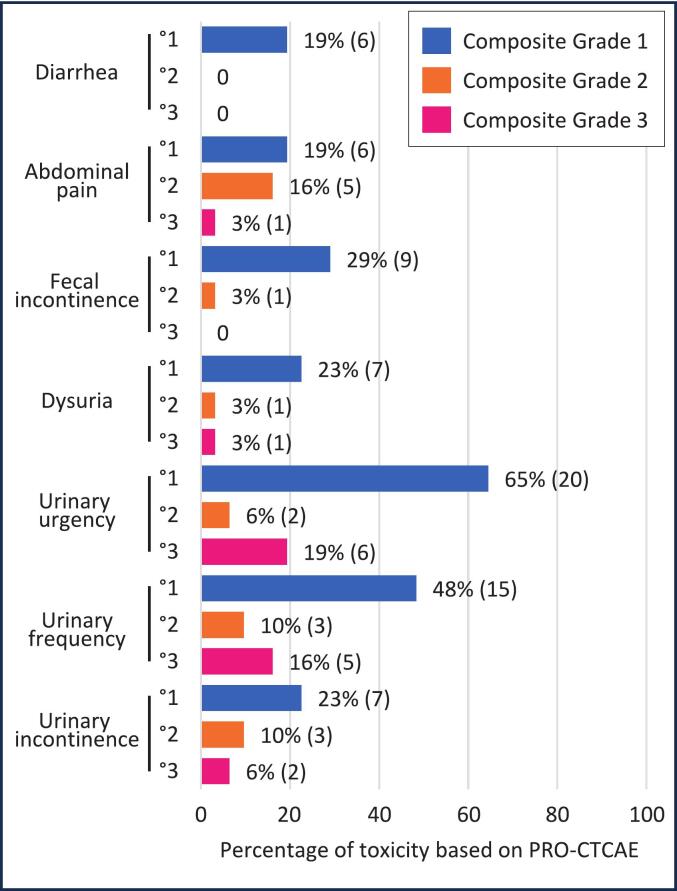
Toxicity at survey of patients (n = 31) after reirradiation of the primary site as measured by PRO-CTCAE composite grades.

Patient-reported toxicity per PRO-CTCAE at time of survey and in the total cohort is shown in Fig. 2. Most patients reported either no toxicity (composite grade 0) or mild toxicity (composite grade 1). PRO-CTCAE composite grade 3 toxicity was reported by 3 % (1/31), 19 % (6/31), 16 % (5/31), and 6 % (2/31) of the patients for painful urination, urinary urgency, urinary frequency, and urinary incontinence, respectively. There was no rectal composite grade 3 toxicity. Supplementary Table S3 shows PRO-CTCAE composite grades by HDR-BT reirradiation and SBRT reirradiation. Three patients reported interventions or complications after reirradiation potentially linked to toxicity of reirradiation including need of a foley catheter (n = 1), infection of a urethral prosthesis (n = 1), and need for transurethral prostate resection (n = 1).

### 3.4 Prevalence of decision regret

**Fig. 3:**
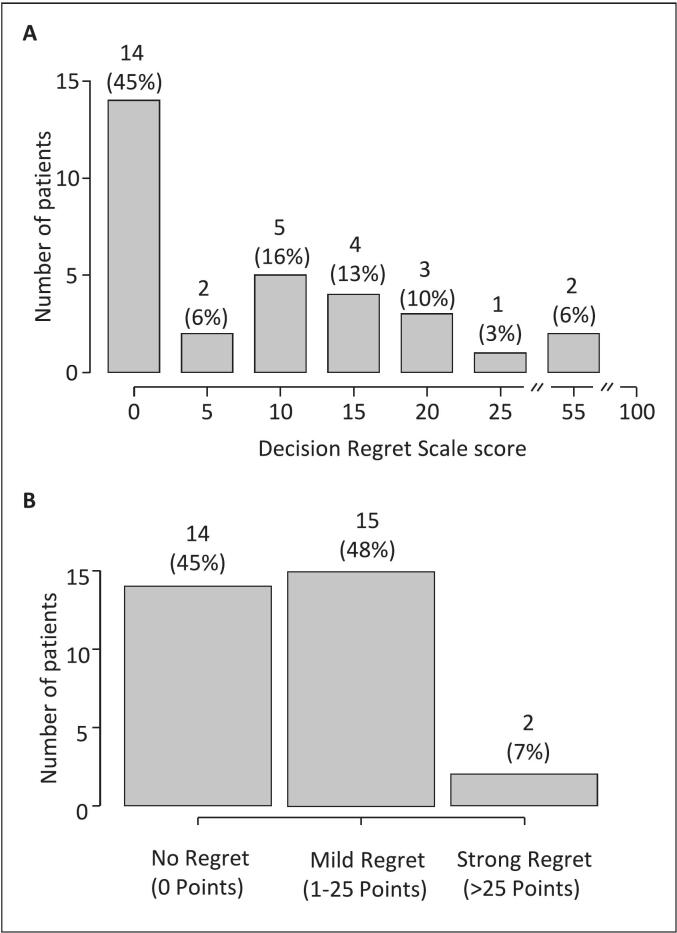
Decision regret among prostate cancer patients (n = 31) after reirradiation of the primary site as per Decision Regret Scale. Panel A displays the distribution of the Decision Regret Scale score (range: 0–100; maximum value in our cohort: 55). Panel B depicts categories of no regret (0 points), mild regret (1–25 points), and strong regret (>25 points).

The distribution of decision regret as per DRS at the time of the survey and in the whole cohort is shown in Fig. 3 Panel A. The mean total DRS score was 10 (SD: 14) and the median total DRS was 5 (IQR: 15). The maximum value was 55 points. No (0 points), mild (1–25 points), or strong regret (>25 points) was reported by 45 % (14/31), 48 % (15/31), and 7 % (2/31) of the patients, respectively. Results of single DRS items are shown in Supplementary Table S4.

### 3.5 Determinants of decision regret

Decision regret (total score) was not significantly associated with non-continuous covariables based on one-way ANOVA analysis as shown in Supplementary Table S5. These covariables included performance status at reirradiation, primary radiotherapy vs radical prostatectomy as initial treatment, HDR-BT vs. SBRT as type of reirradiation, use of ADT at reirradiation, local relapse after reirradiation, and any progression after reirradiation.

To explore associations of decision regret (total score) with further covariables, Pearson’s correlation analysis was used (Table 3). There were significant associations of higher decision regret and lower patient age at survey. Furthermore, there significant associations of higher decision regret with patient reported outcomes including worse EPIC-26 urinary irritative/obstructive, worse EORTC QLQ-C30 social functioning, and higher EORTC QLQ-C30 pain. Decision regret was also significantly associated with worse levels of shared decision making based on question 3 of the PSCC and worse patient satisfaction based on the PSCC summary score. Finally in terms of patient-reported toxicity, worse PRO-CTCAE painful urination and worse PRO-CTCAE urinary frequence were significantly associated with higher decision regret.

**Table 3:** Association of decision regret per Decision Regret Scale and independent variables per Pearson’s correlation after reirradiation to the primary site for prostate cancer (n = 31).

**Decision Regret −**	**N**	**Pearson’s r**	**p**	**Lower 95 % CI**	**Upper 95 % CI**
Age at reirradiation	31	−0.341	0.061	−0.620	0.015
Age at survey	31	−0.424	**0.018**	−0.677	−0.082
Number of comorbidities at survey	31	0.118	0.527	−0.247	0.453
Interval initial radiotherapy until reirradiation	31	−0.019	0.918	−0–371	0.337
Interval reirradiation until survey	31	−0.299	0.102	−0.591	0.062
Urinary incontinence – EPIC 26	31	−0.171	0.359	−0.495	0.196
Urinary irritative/obstructive – EPIC 26	31	−0.398	**0.026**	−0.660	−0.051
Urinary overall function – EPIC 26	31	−0.292	0.111	−0.586	0.069
Bowel function – EPIC 26	31	0.280	0.127	−0.082	0.577
Hormonal function – EPIC 26	31	−0.264	0.152	−0.565	0.100
Sexual function – EPIC 26	31	−0.165	0.374	−0.491	0.201
Physical functioning – EORTC QLQ-C30	31	−0.254	0.168	−0.558	0.111
Social functioning – EORTC QLQ-C30	31	−0.594	**0<.001**	−0.783	−0.304
Emotional functioning – EORTC QLQ-C30	31	−0.315	0.084	−0.602	0.044
Pain – EORTC QLQ-C30	31	0.429	**0.016**	0.088	0.680
Fatigue – EORTC QLQ-C30	31	0.276	0.133	−0.087	0.574
Global HRQoL – EORTC QLQ-C30	31	−0.219	0.236	−0.532	0.147
Shared decision making – PSCC	31	−0.401	**0.025**	−0.662	−0.055
Patient satisfaction – PSCC	27	−0.597	**0.001**	−0.796	−0.281
Diarrhoea – PRO-CTCAE*	31	−0.093	0.620	−0.433	0.270
Abdominal Pain – PRO-CTCAE*	31	0.336	0.065	−0.021	0.617
Faecal incontinence – PRO-CTCAE*	29	−0.248	0.195	−0.563	0.131
Painful urination – PRO-CTCAE*	31	0.418	**0.019**	0.074	0.672
Urinary urgency – PRO-CTCAE*	31	0.212	0.252	−0.154	0.527
Urinary frequency – PRO-CTCAE*	29	0.402	**0.031**	0.042	0.670
Urinary incontinence – PRO-CTCAE*	31	0.132	0.480	−0.233	0.464

*Composite grading according to: Basch, E., Becker, C., Rogak, L.J., Schrag, D., Reeve, B.B., Spears, P., Smith, M.L., Gounder, M.M., Mahoney, M.R., Schwartz, G.K., Bennett, A.V., Mendoza, T.R., Cleeland, C.S., Sloan, J.A., Bruner, D.W., Schwab, G., Atkinson, T.M., Thanarajasingam, G., Bertagnolli, M.M., Dueck, A.C., 2021. Composite Grading Algorithm for the National Cancer Institute’s Patient-Reported Outcomes version of the Common Terminology Criteria for Adverse Events (PRO-CTCAE). Clin Trials 18, 104–114.https://doi.org/10.1177/1740774520975120.

Abbreviations: EPIC-26, Expanded prostate cancer index composite; EORTC QLQ-C30, European Organization for Research and Treatment of Cancer Quality of Life Core Questionnaire; PSCC, Patient Satisfaction with Cancer-related Care.

## 4 Discussion

In this bi-centre cross-sectional study, we assessed the prevalence and potential determinants of decision regret among 31 prostate cancer patients after reirradiation of the primary site via HDR-BT or SBRT. The prevalence of decision regret was low to moderate overall and patient-reported outcomes emerged as determinants of decision regret. To our knowledge, this is the first study to report on decision regret from the patient’s perspective after reirradiation for prostate cancer as well as for any other cancer overall.

Characteristics of our study cohort may first be put in context of previously published cohorts of reirradiation for radio-recurrent prostate cancer. Valle and colleagues conducted a systematic review and *meta*-analysis of studies on local salvage therapy, including HDR-BT and SBRT approaches [5]. Although patient characteristics of studies in this review and our cohort were mostly similar, some differences exist: patients were slightly older (75 years vs. 71 years for HDR-BT and 72 years for SBRT), had higher PSA-values prior to reirradiation (6.3 ng/ml vs. 4.5 ng/ml for HDR-BT and 4.0 ng/ml for SBRT), longer time intervals from initial therapy to reirradiation (8 years vs. 5.1 years for HDR-BT and 7.3 years for SBRT), and less often confirmatory biopsy prior to reirradiation (26 % vs. 94 % for HDR-BT and 81 % for SBRT) in our cohort. Oncological outcomes fell into the range of our cohort as 2-year recurrence-free survival was reported to be 77 % for HDR-BT and 62 % for SBRT, respectively [5]. The review reported severe genitourinary toxicity, mostly based on CTCAE grade ≥ 3, as 8 % for HDR-BT and 4.2 % for SBRT, respectively [5]. Different domains of genitourinary toxicity of grade 3 fell into the range of 3 % to 19 % of the patients in our cohort. While this rate may appear more pronounced as compared to the literature at first, one should note differences in toxicity reporting (PRO-CTCAE in our cohort vs. CTCAE in the literature) and the well-advanced median age at survey (81 years) in our cohort.

The prevalence of decision regret in our cohort amounted to a mean DRS value of 10 and 7 % of the patients reported strong regret. Becerra Pérez and colleagues reported in a systematic review of decision regret among patients with various health conditions a mean DRS value of 16.5 [9]. Furthermore, a recent systematic review and *meta*-analysis including 17,883 prostate cancer patients reported a rate of significant decision regret of 20 % after initial therapy [22]. Rühle and colleagues found a mean DRS value of 14 and a proportion of 18 % reporting strong decision regret among 207 patients undergoing radiotherapy for various cancer types [13]. Finally, a study from our group on long-term decision regret among 108 prostate cancer patients after initial treatment with EBRT and HDR-BT boost found a DRS mean value of 11 and 12 % of the patients reported strong regret [11]. Taken together, the prevalence of decision regret after reirradiation for radio-recurrent prostate cancer appears to be relatively mild and potentially even lower than in patients at initial treatment based on the available literature. This finding is particularly relevant, as guidance from randomized studies is lacking so far for patients with localized radio-recurrent disease.

Determinants of decision regret were mainly patient-reported outcomes. Patients with higher urinary symptom and toxicity burden reported higher decision regret. This finding is well in line with our previous study on prostate cancer patients after initial EBRT and HDR-BT boost [11]. Furthermore, previous observational studies and systematic reviews highlighted the negative association of post-treatment symptom burden and decision regret [9,10,[23], [24], [25], [26], [27]].

Interestingly, lower levels of shared-decision making and patient satisfaction were also associated with stronger decision regret in our cohort. This has been described before in patients with prostate cancer after initial therapy, but also in patients with diverse health conditions [9,13,24,28,29]. Our finding has important implications on decision making before local salvage therapy for radio-recurrent prostate cancer as there are no clear guidelines on which treatment approach to choose. Careful and nuanced shared-decision making is therefore crucial and should include a thorough discussion on potential toxicity but also oncological outcomes. However, oncological outcomes (biochemical relapse, local relapse, distant relapse, and PFS) were not associated with decision regret in our cohort. While this may appear counterintuitive at first, the impact of recurrence after initial treatment on decision regret is unclear in the current literature [13,23,24].

Our study has limitations. Though we report the first cohort with decision regret data after reirradiation, the sample size is small and characteristics of reirradiation were heterogenous. This does not allow for causal conclusions, especially not when comparing HDR-BT to SBRT for reirradiation. Next, our cohort displayed some differences from other published cohorts on reirradiation for radio-recurrent prostate cancer as described above such as rates of confirmatory biopsy prior to reirradiation. This may reduce the external validity of our exploratory findings. Finally, the effect size of associations of determinants on decision regret was weak to moderate.

## 5 Conclusions

In conclusion, decision regret was mild after reirradiation of the primary site in prostate cancer patients. Decision regret may be associated with patient-reported urinary symptoms and toxicity. Furthermore, patient satisfaction and shared-decision making could impact levels of decision regret. These findings fill an evidence gap in the context of reirradiation. After further validation, they may be useful when making treatment decisions together with patients prior to local salvage therapy of radio-recurrent disease.

## Declaration of Competing Interest

The authors declare that they have no known competing financial interests or personal relationships that could have appeared to influence the work reported in this paper.

AF has received honoraria from Merck Sharp &Dohme and a research award from Lilly Deutschland Stiftung.DK has received honoraria from Astra Zeneca, best practice onkologie, ESO, ESMO, Gilead, med update, Merck Sharp & Dohme, Novartis, onkowissen, and Pfizer, as well as research funding from Stiftung Deutsche Krebshilfe and Merck KGaA.

OW received honoraria and travel grants from Brainlab AG and novocure AG.SR has received honoraria from BMS, Merck, MSD and Novartis and has equity interest in Rocketlane Medical Ventures GmbH.

All other authors declare no conflicts of interest.
