# Supplementary material for: Decision regret after reirradiation of the primary site in patients with prostate cancer
Source: Clin Transl Radiat Oncol. 2025 Jul 19;54:101019. doi: 10.1016/j.ctro.2025.101019 (PMC12320099; doi:10.1016/j.ctro.2025.101019)
Supplement: Supplementary Data 1 [file mmc1.pdf]

# Decision regret after re-irradiation of the primary site in patients with prostate cancer

Corresponding author: Alexander Fabian, MD (alexander.fabian@uksh.de)
Department of Radiation Oncology, University Hospital Schleswig-Holstein Campus Kiel, Kiel, Germany, Arnold-Heller-Str.3, 24105 Kiel, Tel. +49-431-500-26501; Fax +49-431-500-26548


| | |
|---|---|
| **Supplementary Figure 1** Study Flow Chart | p2 |
| **Supplementary Table 1** Patient characteristics at survey | p3 |
| **Supplementary Table 2** Selected patient-reported outcomes at survey of prostate cancer patients (n = 31) with re-irradiation to the primary site | p3 |
| **Supplementary Table 3** Patient-reported (n = 31) toxicity at survey based on PRO-CTCAE | p4 |
| **Supplementary Table 4** Results of single items of the Decision Regret Scale (DRS) (n = 31) | p4 |
| **Supplementary Table 5** Association of decision regret per Decision Regret Scale and non-continuous independent variables per one-way ANOVA after re-irradiation to the primary site for prostate cancer (n = 31) | p5 |

Supplementary material

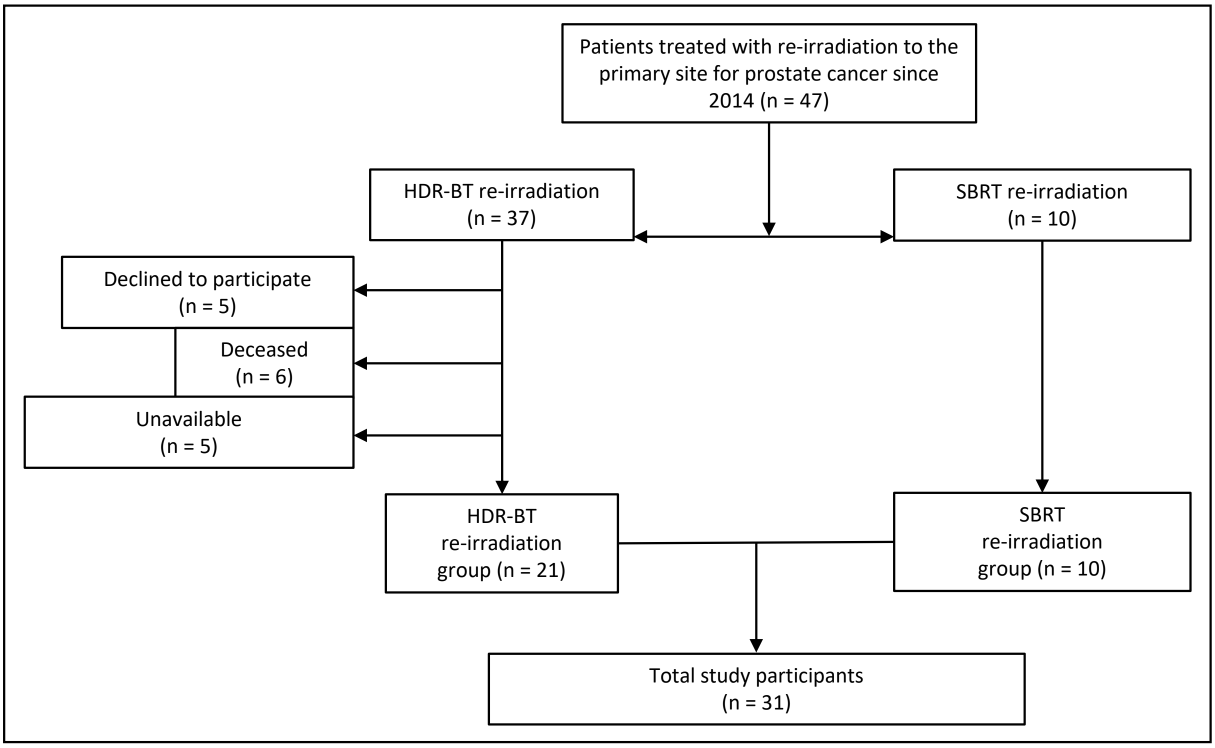


**Supplementary Figure 1** Study Flow Chart. Abbreviations: HDR-BT, high dose-rate brachytherapy; SBRT, stereotactic body radiotherapy

**Supplementary Table 1** Patient characteristics at survey

| | | HDR-BT re-irradiation group 68% (n=21) | SBRT re-irradiation group 32% (n=10) | Total 100% (31) |
|---|---|---|---|---|
| Age (years) | | Median: 81; IQR: 9 | Median: 80; IQR: 16 | Median: 81; IQR: 9 |
| Comorbidity | SCQ (score represents number of comorbidities) | Median: 5; IQR: 7 | Median: 5; IQR: 4 | Median: 5; IQR: 5 |
| Global HRQoL | EORTC QLQ-C30 (higher HRQoL with higher values on a range 0-100) | Mean: 61; SD: 29 | Mean: 73; SD: 8 | Mean: 65; SD: 25 |

Abbreviations: EORTC QLQ-C30, European Organization for Research and Treatment of Cancer Quality of Life Core Questionnaire, SCQ, Self-Administered Comorbidity Questionnaire

**Supplementary Table 2** Selected patient-reported outcomes at survey of prostate cancer patients (n = 31) with re-irradiation to the primary site

| Domain | Questionnaire | Mean | Standard deviation |
|---|---|---|---|
| Urinary incontinence | EPIC-26 | 76 | 28 |
| Urinary irritative/obstructive | EPIC-26 | 81 | 19 |
| Urinary overall function | EPIC-26 | 73 | 30 |
| Bowel function | EPIC-26 | 91 | 14 |
| Hormonal function | EPIC-26 | 84 | 16 |
| Sexual function | EPIC-26 | 21 | 15 |
| Physical functioning | EORTC QLQ-C30 | 77 | 25 |
| Social functioning | EORTC QLQ-C30 | 74 | 32 |
| Emotional functioning | EORTC QLQ-C30 | 79 | 26 |
| Pain | EORTC QLQ-C30 | 22 | 30 |
| Fatigue | EORTC QLQ-C30 | 30 | 25 |
| Shared decision making | PSCC | 90 | 18 |
| Patient satisfaction | PSCC | 90 | 11 |

EPIC-26 domains show better functioning with higher scores. EORTC QLQ-C30 domains show better functioning with higher function scores, but worse symptoms with higher symptom scores. PSCC scores show higher satisfaction with higher values. Abbreviations: EPIC-26, Expanded prostate cancer index composite; EORTC QLQ-C30, European Organization for Research and Treatment of Cancer Quality of Life Core Questionnaire; PSCC, Patient Satisfaction with Cancer-related Care

**Supplementary Table 3** Patient-reported (n = 31) toxicity at survey based on PRO-CTCAE

| PRO-CTCAE domain | PRO-CTCAE composite grade* | HDR-BT re-irradiation group 68% (n=21) | SBRT re-irradiation group 32% (n=10) | Total 100% (n=31) |
|---|---|---|---|---|
| Diarrhea | °1 | 19% (4) | 20% (2) | 19% (6) |
| | °2 | 0 | 0 | 0 |
| | °3 | 0 | 0 | 0 |
| Abdominal Pain | °1 | 19% (4) | 20% (2) | 19% (6) |
| | °2 | 10% (2) | 30% (3) | 16% (5) |
| | °3 | 5% (1) | 0 | 3% (1) |
| Fecal incontinence | °1 | 24% (5) | 40% (4) | 29% (9) |
| | °2 | 5% (1) | 0 | 3% (1) |
| | °3 | 0 | 0 | 0 |
| Painful urination | °1 | 19% (4) | 30% (3) | 23% (7) |
| | °2 | 5% (1) | 0 | 3% (1) |
| | °3 | 0 | 10% (1) | 3% (1) |
| Urinary urgency | °1 | 62% (13) | 70% (7) | 65% (20) |
| | °2 | 10% (2) | 0 | 6% (2) |
| | °3 | 24% (5) | 10% (1) | 19% (6) |
| Urinary frequency | °1 | 43% (9) | 60% (6) | 48% (15) |
| | °2 | 14% (3) | 0 | 10% (3) |
| | °3 | 19% (4) | 10% (1) | 16% (5) |
| Urinary incontinence | °1 | 29% (6) | 10% (1) | 23% (7) |
| | °2 | 10% (2) | 10% (1) | 10% (3) |
| | °3 | 10% (2) | 0 | 6% (2) |

*Composite grading according to: Basch, E., Becker, C., Rogak, L.J., Schrag, D., Reeve, B.B., Spears, P., Smith, M.L., Gounder, M.M., Mahoney, M.R., Schwartz, G.K., Bennett, A.V., Mendoza, T.R., Cleeland, C.S., Sloan, J.A., Bruner, D.W., Schwab, G., Atkinson, T.M., Thanarajasingam, G., Bertagnolli, M.M., Dueck, A.C., 2021. Composite Grading Algorithm for the National Cancer Institute's Patient-Reported Outcomes version of the Common Terminology Criteria for Adverse Events (PRO-CTCAE). Clin Trials 18, 104–114. https://doi.org/10.1177/1740774520975120
Abbreviation: PRO-CTCAE, Patient-reported Outcome Version of the Common Terminology Criteria of Adverse Events

**Supplementary Table 4** Results of single items of the Decision Regret Scale (DRS) (n = 31)

| Item No. | Item wording | Mean | SD | Range |
|---|---|---|---|---|
| 1 | It was the right decision | 5 | 14 | 0-50 |
| 2 | I regret the choice that was made | 2 | 10 | 0-50 |
| 3 | I would go for the same choice if I had to do it over again | 5 | 15 | 0-50 |
| 4 | The choice did me a lot of harm | 34 | 37 | 0-100 |
| 5 | The decision was a wise one | 5 | 14 | 0-50 |
| | Total Score | 10 | 14 | 0-55 |

Higher means on single items indicate higher agreement (range: 0-100)
A higher mean on the total score indicates higher decision regret (range: 0-100)

**Supplementary Table 5** Association of decision regret per Decision Regret Scale and non-continuous independent variables per one-way ANOVA after re-irradiation to the primary site for prostate cancer (n = 31)

| **Variable** | **N** | **Mean** | **SD** | **p** |
|---|---|---|---|---|
| Performance status at re-irradiation | | | | 0.5 |
| ECOG 0 | 5 | 6 | 8.9 | |
| ECOG 1 | 21 | 9.8 | 12.6 | |
| ECOG 2 | 5 | 16 | 24.3 | |
| Initial treatment | | | | 0.6 |
| Primary radiotherapy | 25 | 9.6 | 15.2 | |
| Primary radical prostatectomy | 6 | 12.5 | 9.9 | |
| Type of re-irradiation | | | | 0.8 |
| HDR-BT | 21 | 9.8 | 13.3 | |
| SBRT | 10 | 11 | 17.0 | |
| ADT at re-irradiation | | | | 0.8 |
| Yes | 8 | 11.3 | 19.4 | |
| No | 23 | 9.8 | 12.6 | |
| Local relapse after re-irradiation | | | | 0.7 |
| Yes | 10 | 9.5 | 17.2 | |
| No | 15 | 12 | 14.6 | |
| Any progression* after re-irradiation | | | | 0.7 |
| Yes | 17 | 9.1 | 14.1 | |
| No | 14 | 11.4 | 15.0 | |

* Biochemical relapse, local relapse, or distant relapse
Abbreviations: ADT, androgen deprivation therapy; HDR-BT, high dose-rate brachytherapy; SBRT, stereotactic body radiotherapy
